# Supplementary material for: The role of the pigment–protein complex LHCBM1 in nonphotochemical quenching in Chlamydomonas reinhardtii
Source: Plant Physiol. 2023 Oct 17;194(2):936–44. doi: 10.1093/plphys/kiad555 (PMC10828212; doi:10.1093/plphys/kiad555)
Supplement: kiad555_Supplementary_Data [file kiad555_supplementary_data.zip › PP2023RA00234DR1_Supplemental_Data.pdf]

Supplemental data

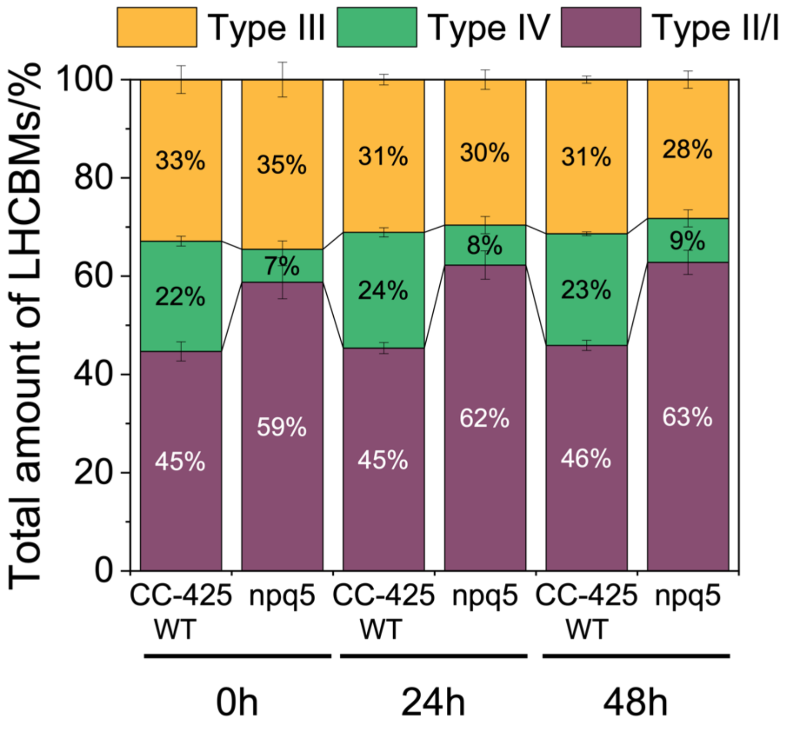

Supplemental Figure S1. Quantification of LHCBM subtypes based on Immunoblotting during HL treatment in WT (CC-425) and npq5. Densitometry data of each subtype were normalized to the total amount of LHCBMs in each strain at each time point. Data shown are mean ± SEM, n=3 (WT CC-425) or 8 (npq5) biological replicates.

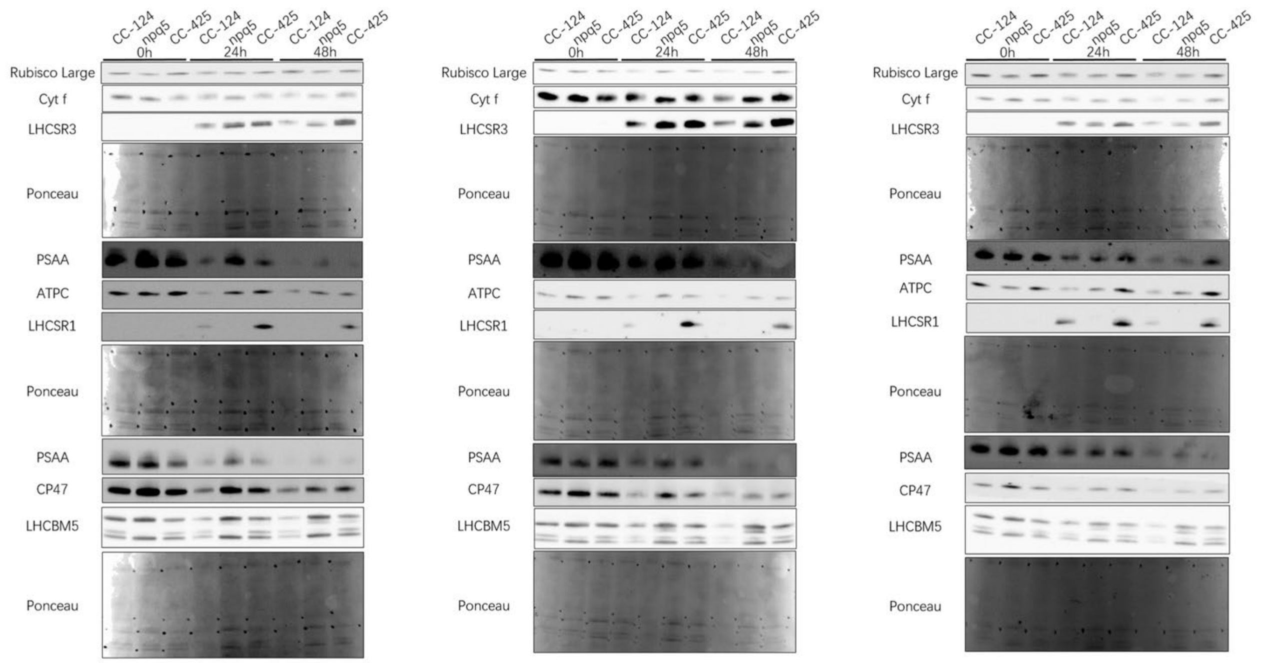

Supplemental Figure S2. Immunoblots of photosynthetic proteins during HL in WT (CC-124 and CC-425) and npq5. 5 µg total proteins were loaded in each well. Each panel shows one biological replicates.

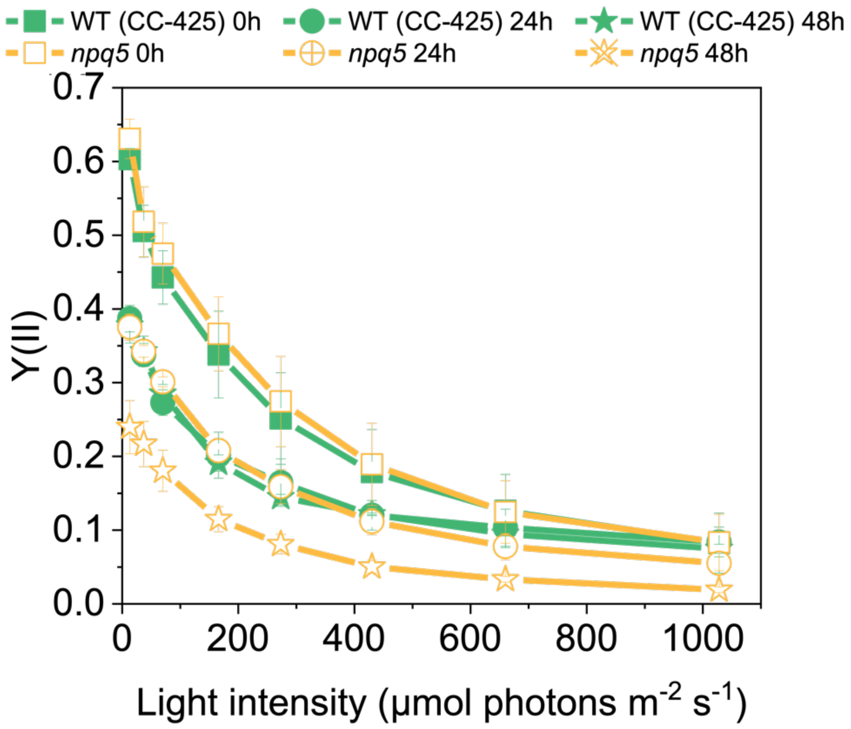

Supplemental Figure S3. Photosystem II efficiency Y(II) measured at different light intensities. Data shown are mean ± SEM, n=3 biological replicates.

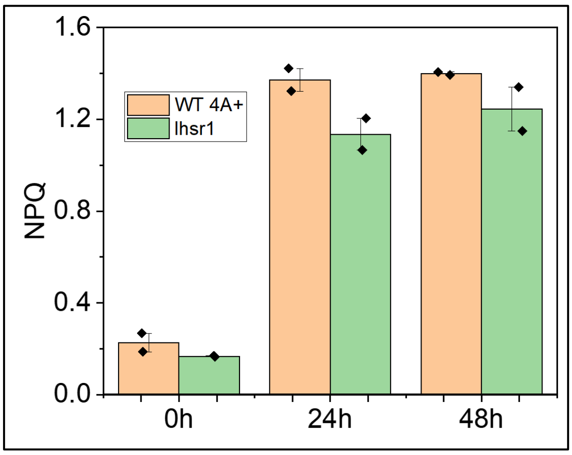

Supplemental Figure S4. Maximum NPQ in WT (4A+) and lhcsr1 during HL treatment. The data are the result of two biological replicates.

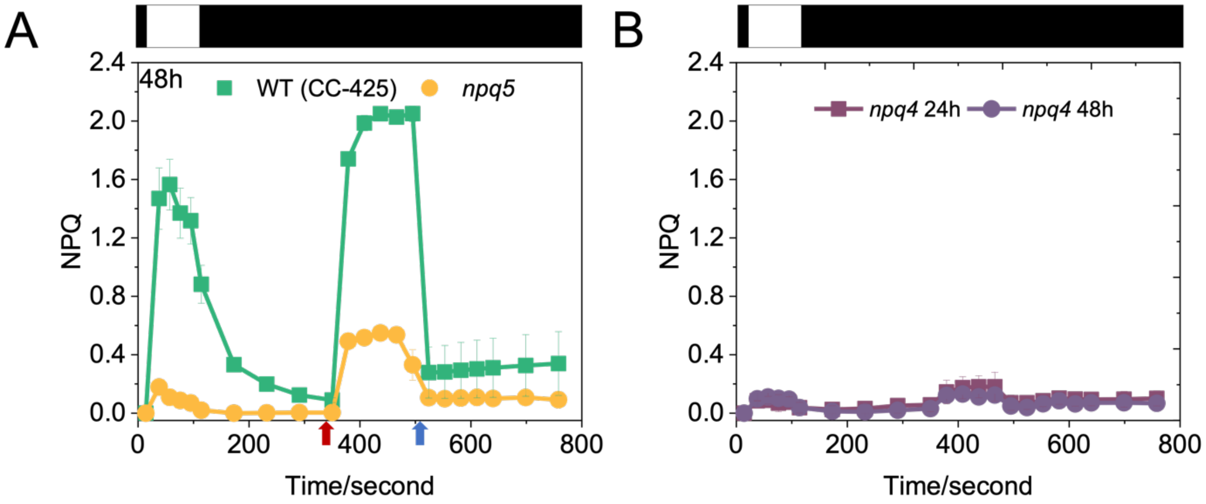

Supplemental Figure S5. Non-photochemical quenching measurements. Comparison of light-induced and acid-induced NPQ in npq5 and WT (CC-425) at 48 h (A) and npq4 during HL (B). The additions of 1 M acetic acid (decreases pH to 5.5) and 2 M KOH (neutralizes pH to 7.0) in (A) are indicated by red and blue arrows, respectively. Illumination (1500 µmol photons m^{-2} s^{-1}) and dark phases are indicated by white and black bars, respectively. Data shown are mean ± SE, n=3 (WT CC-425) and n=5 (npq5) biological replicates in (A) and n=2 in (B).

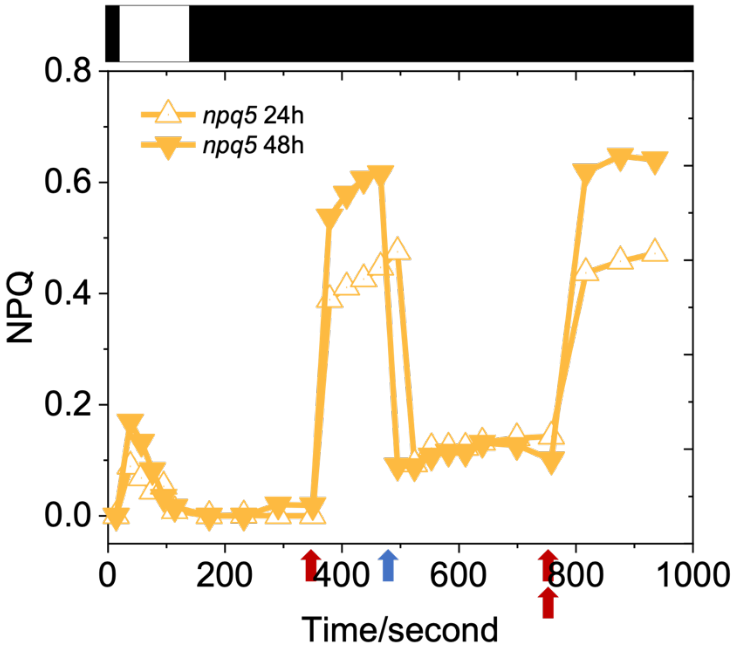

Supplemental Figure S6. NPQ induction with acid. Double amount of 1 M acetic acid addition in npq5. The white and black bars indicate the illumination and recovery in darkness, respectively. The addition of acid or base is indicated by red or blue arrows, respectively. Two-red arrows indicate the double amount of acid added in the cell culture.
