# Supplementary material for: The role of the pigment–protein complex LHCBM1 in nonphotochemical quenching in Chlamydomonas reinhardtii
Source: Plant Physiol. 2023 Oct 17;194(2):936–44. doi: 10.1093/plphys/kiad555 (PMC10828212; doi:10.1093/plphys/kiad555)
Supplement: kiad555_Supplementary_Data [file PP2023RA00234DR1_Merged_Decision_Summary_PDF.pdf › PP2023RA00234DR1_Merged_Decision_Summary_PDF.pdf]

Plant Physiology®

LHCBM1 tunes the response of LHCSR3-dependent non-photochemical quenching in Chlamydomonas

Xin Liu, Wojciech Nawrocki, and Roberta Croce

Review Timeline:

Submission Date:	20-Jan-2022
Editorial Decision:	15-Feb-2022
Resubmission Received:	17-Feb-2023
Editorial Decision:	28-Apr-2023
Revision Received:	21-Sep-2023
Accepted:	23-Sep-2023

15-Feb-2022

Prof. Dr. Roberta Croce
Vrije Universiteit Amsterdam
Amsterdam, Netherlands 1081 HV
Netherlands

RE: The role of LHCBM1 in non-photochemical quenching in Chlamydomonas reinhardtii

Dear Dr. Roberta Croce:

Thank you for giving us the opportunity to review your manuscript for publication in the Plant Physiology. The reviewers have found the manuscript of interest, but have raised a number of concerns that will require your attention. As these concerns are of some gravity and will entail substantial changes to the manuscript, you are likely to need additional time to address them. I must therefore reject the manuscript in its present form. Nonetheless, if you are able to address the points raised in review, I would welcome your resubmission of a manuscript that incorporates your responses.

In particular, I direct your attention to the following comments for consideration in a resubmitted manuscript. Both reviewer commented that further data are needed to support the Western blot analyses and suggested the inclusion of further biophysical experiments to support the conclusions drawn. They also raised concerns over the mutant used and suggested including further work using the npq5 complement that is available. One reviewer also felt that the paper contains a number of unexplained data/conclusions that were not related to the main conclusions and either needed to experimentally supported or removed from the MS.

Please note that any resubmission will be treated as a new entry to the system and is likely to go out for review.

If you do have any queries or concerns, you are welcome to contact me in the first instance.

Thank you again for allowing Plant Physiology to review your work. We look forward to hearing from you soon.

Sincerely,
Plant Physiology Board of Editors

-- Reviewer comments:
Reviewer #1 (Comments for the Author):

This paper (Liu et al.) addresses the question concerning the role of LHCBM1 in non-photochemical quenching (NPQ) in Chlamydomonas reinhardtii. The authors use mutant cells lacking this protein and apply conventional Chl-a fluorescence transient measurements to determine the light- and low-pH induced NPQ in LL and HL grown cells; they also apply electrochromic absorbance transient measurements to deterermine the PSI/PSII ratios as well as immunoblot protein analyis. The authors arrive to the conclusion that LHCBM1 participates in NPQ via interacting with the LHCSR3. I think it is an important conclusion which is supported mainly by NPQ measurements - which should eventually be reported in Plant Physiol. However, the paper also contains quite a number of unexplained data and conclusions (which, in my view, do not concern their main conclusion) which need further experimental support (or should be dropped).
1/ The authors claim (p. 6) that "The cells respond to the reduced PSII functionality by lowering the amount of PSI to restore a balance between the two photosystems." It is unclear if they propose an active mechanism or PSI is also damaged? Also, more importantly, such a statement should be substantiated either by ECS measurements (on Chl basis) - e.g testing the initial aplitude with and without far red background illumination. P700 measurements could also be performed.
2/ The data in Fig. S2 are odd: (i) I assume that the middle lanes are from 24 h HL; (ii) PSAA in some cases simply disappears, which - if true - should have severe consequences. This should be explained. Can you rule out any systematic technical problem of immuno-detecting PSI from HL-treated cells? .(iii) I wonder how these data could be used for quantitative analysis. Again, shoudl be substantiated (or replaced?) by functional measurements (P700, deltaA(ECS), or CO_2 fixation, full-chain electron transport measurements etc. (iv) Concerning the PSAA/CP47 ratio (beyond all the uncertainities above) can these data be reconciled by assuming that a fraction of CP47 comes from damaged PSII?
3/ Additional remarks:
(i) The Fv/Fm ratio cannot and should not be equated with the maximum quantum efficiency of PSII photochemistry. (See Sipka et al. 2021 Plant Cell)

(ii) Fig. 1B: incorrect X-axis label
(iii) Fig. 4: X-axis label should be Light intensity or Photon flux density (in singular)
(iv) Add spaces, where needed before the unit of tzhe dimension (e.g. 0h - 0 h)
(v) supposting - supporting; and make clear which hypothesis you mean

Reviewer #2 (Comments for the Author):

During light fluctuations, photosynthesis has to acclimate dynamically to various levels of light intensities and protect itself from harmful light energy. One of the critical processes is the non-photochemical quenching, allowing safe dissipation of absorbed light energy, which in Chlamydomonas is mostly mediated by the protein LHCSR3. While in Chlamydomonas, the mutant of the protein LHCBM1 has been also identified as a key NPQ component, it is not the direct quencher of the light energy and it's role has remained puzzling.
Here, Liu et al confirm that the mutant of LHCBM1 (npq5) is lacking NPQ. They show that this is not due to the lack of LHCSR3 accumulation. They further show that lower NPQ in npq5 is rather due to a lower connectivity of the light harvesting antennae. Based on the literature they thus propose that LHCBM1 role in NPQ could be in interacting with LHCRS3, stabilizing it's quenching state.
The manuscript is well written and the logic easy to follow, I particularly thank the authors for having embedded their figures in the main text, which helps the reviewing process. My main concern here is that all conclusions are based on the analysis of one strain (npq5) that have been produced nearly 30 years from now and (to the best of my knowledge), kept on plates in the laboratory of Pr. Niyogi since then. Chlamydomonas strains are known to accumulate mutations over time that tend to reverse phenotypes (www.plantcell.org/cgi/doi/10.1105/tpc.15.00508). I this context, I think it would be necessary to also analyse alongside npq5, at least one complementing strain or another independent lhcbm1 mutant. This would help in revealing what effects are linked with the mutation and strengthen the authors conclusions.
Major remarks:
1- The method section is rather limited in explanations and description of the experimental procedure. The manuscript would gain a lot in clarity of the experimental procedures by elaborating more on what has been done, especially for quantification of proteins and quantifying the PSII absorbance cross section.
2- A lot of emphasis is put on protein quantification using western blot. However, such method has to be analysed with a lot of cautious and require many controls that are not shown in the present manuscript. For example, normalizing to an internal loading control (like actin or tubulin) should have been done; absence of saturation of the signal should have been ensured... Clarification of the western blot-based quantification would help in this manner. Please provide raw data with molecular weight ladder if possible to improve reproducibility of the results.
3- The title is a bit misleading here since it gives the impression that the authors have revealed the mechanistic role of LHCBM1. While they only propose a mechanism for LHCBM1 role, they have shown that lack of NPQ in npq5 is not due to low LHCSR3 amount but rather to its capacity to respond to pH. Thus a title like "LHCBM1 tunes the proton response of LHCSR3-mediated NPQ" would be more appropriate.

Minor remarks:

The authors show that the level of LHCSR1 is strongly impacted in npq5 mutant, it would be nice to have the authors' view on this effect in the discussion. Could the lack of LHCBM1 completely destabilize LHCSR1 protein and lead to its degradation? Would LHCBM1 also be the main docking site of LHCSR1?

Please add lines number to facilitate remarks.

"A quickly activated photoprotection mechanism": NPQ is not necessarily quickly activated. Please consider revising

" expressed upon exposure of the cells to high light": LHCSR3 has recently been shown to be also importantly controlled by CO2 levels, which presumably would be the main factor (https://doi.org/10.1101/2021.10.23.465040) , please also consider citing more recent analysis like (https://doi.org/10.1101/2021.10.23.463292).

At some places, the authors are using "*" to separate between units, like "photons*m-2
s-1"; please consider changing the "" by a space, like in Fig. 2C. Please check that the formatting used in consistent throughout the manuscript.

When showing histograms or bar plots, please show explicitly each biological replicate, since for small data sets (n<10), it is easier to visualize for the reader and more transparent than +-SEM or +-SD.
Please also provide statistical analysis on the data whenever possible

"lower PSI/PSII ratio than the control strain.": Please provide a reference to a figure here

"The cells respond to the reduced PSII

functionality by lowering the amount of PSI to restore a balance between the two photosystems.": Please provide a reference to a figure here

Response to the reviewers' comments

Reviewer 1

This paper (Liu et al.) addresses the question concerning the role of LHCBM1 in non-photochemical quenching (NPQ) in Chlamydomonas reinhardtii. The authors use mutant cells lacking this protein and apply conventional Chl-a fluorescence transient measurements to determine the light- and low-pH induced NPQ in LL and HL grown cells; they also apply electrochromic absorbance transient measurements to deterermine the PSI/PSII ratios as well as immunoblot protein analyis.
The authors arrive to the conclusion that LHCBM1 participates in NPQ via interacting with the LHCSR3. I think it is an important conclusion which is supported mainly by NPQ measurements - which should eventually be reported in Plant Physiol. However, the paper also contains quite a number of unexplained data and conclusions (which, in my view, do not concern their main conclusion) which need further experimental support (or should be dropped).
Answer: are glad the reviewer finds our work interesting and we thank them for their help in improving the manuscript. We have now revised the manuscript according to the suggestions.

1/ The authors claim (p. 6) that "The cells respond to the reduced PSII functionality by lowering the amount of PSI to restore a balance between the two photosystems." It is unclear if they propose an active mechanism or PSI is also damaged? Also, more importantly, such a statement should be substantiated either by ECS measurements (on Chl basis) - e.g testing the initial aplitude with and without far red background illumination. P700 measurements could also be performed.
Answer: We have deleted the sentence. Indeed, it is a challenging task to disentangle the downregulation of PSI from its increased degradation, and we do not attempt to do it in this work. We note that the P700 measurements allow probing the redox status of the donor- and acceptor sides of active PSI (i.e. those which exhibit long-lived absorption difference signals corresponding to the oxidation and reduction of the P700 chlorophyll pair), but are not suitable to quantify PSI photoinhibition (which influences the amplitude of this redox signal). This is true, particularly in the case of antenna mutants in variable environmental conditions, where the chl content normalization is no longer reliably possible.

2/ The data in Fig. S2 are odd: (i) I assume that the middle lanes are from 24 h HL;
Answer:Thank you. Indeed, those are samples at 24h HL, we have now corrected the figure

(ii) PSAA in some cases simply disappears, which - if true - should have severe consequences. This should be explained. Can you rule out any systematic technical problem of immuno-detecting PSI from HL-treated cells?
Answer: The PSAA signal does not completely disappear, but it is reduced after 48h of high light as is also the case of PSII protein. This corresponds to the (passive or active) adjustment of the PSI:PSII RC ratio discussed above. This is visible in all three biological replicas, which excludes technical problems. The cells are stressed after 48h of HL and this is why our conclusions are mainly based on 24h treatment.

.(iii) I wonder how these data could be used for quantitative analysis. Again, shoudl be substantiated (or replaced?) by functional measurements (P700, deltaA(ECS), or CO_2 fixation, full-chain electron transport measurements etc.
Answer: are also reporting the functional data (ECS data in figure 3a). However, we believe that, a combination of biochemical and functional assays – which we provide in the manuscript - is

necessary to fully understand the functional consequences of light acclimation. This is why we are also reporting the protein data.

(iv) Concerning the PSAA/CP47 ratio (beyond all the uncertainities above) can these data be reconciled by assuming that a fraction of CP47 comes from damaged PSII?
Answer: Yes, this is the conclusion reported on page 6 “These results suggest that part of PSII is not functional in high light and that this population is larger in *npq5* than in the WT (CC-425).”

3/ Additional remarks:
(i) The Fv/Fm ratio cannot and should not be equated with the maximum quantum efficiency of PSII photochemistry. (See Sipka et al. 2021 Plant Cell)
Answer: FV/FM is not the maximum quantum efficiency of PSII (Wienjtes et al., 2012), but if a saturating pulse and not a single-turnover flash is used for the experiments, it is a close proxy of the QE. Sipka et al demonstrate that a single QA reduction event is not sufficient to reach FM, rather than that multi-turnover pulse doesn’t allow obtaining FM.

(ii) Fig. 1B: incorrect X-axis label
Thank you, we have corrected it
(iii) Fig. 4: X-axis label should be Light intensity or Photon flux density (in singular)
Done
(iv) Add spaces, where needed before the unit of tzhe dimension (e.g. 0h - 0 h)
Both are used, we are happy to adapt to the journal style
(v) supposting - supporting; and make clear which hypothesis you mean
Done

Reviewer #2 (Comments for the Author):

During light fluctuations, photosynthesis has to acclimate dynamically to various levels of light intensities and protect itself from harmful light energy. One of the critical processes is the non-photochemical quenching, allowing safe dissipation of absorbed light energy, which in Chlamydomonas is mostly mediated by the protein LHCSR3. While in Chlamydomonas, the mutant of the protein LHCBM1 has been also identified as a key NPQ component, it is not the direct quencher of the light energy and it's role has remained puzzling.
Here, Liu et al confirm that the mutant of LHCBM1 (npq5) is lacking NPQ. They show that this is not due to the lack of LHCSR3 accumulation. They further show that lower NPQ in npq5 is rather due to a lower connectivity of the light harvesting antennae. Based on the literature they thus propose that LHCBM1 role in NPQ could be in interacting with LHCRS3, stabilizing it's quenching state.
The manuscript is well written and the logic easy to follow, I particularly thank the authors for having embedded their figures in the main text, which helps the reviewing process. My main concern here is that all conclusions are based on the analysis of one strain (npq5) that have been produced nearly 30 years from now and (to the best of my knowledge), kept on plates in the laboratory of Pr. Niyogi since then. Chlamydomonas strains are known to accumulate mutations over time that tend to reverse phenotypes (www.plantcell.org/cgi/doi/10.1105/tpc.15.00508). I this context, I think it would be necessary to also analyse alongside npq5, at least one complementing strain or another independent lhcbm1 mutant. This would help in revealing what effects are linked with the mutation and strengthen the authors conclusions.

Answer: We are glad the reviewer appreciated our work and we want to thank them for the helpful suggestions. Unfortunately, the complemented strain is not available. The mutant and the complemented strain were produced in the Grossman lab (Elrad et al 2002), but they do not have the complemented strain anymore. A post-doc of Prof. Grossman tried to make it again, but he was not successful. We also looked for an independent mutant, and we identified only one possible candidate with an insertion in the Lhcbm1 gene, but unfortunately, the analysis of the cells shows that it still contains the protein. Although it was not possible to analyze an independent mutant we are confident that our results do not depend on possible new mutations accumulated in the strain, since the properties of the mutant are identical to those reported by Grossman in the original publication (Elrad et al. 2002). In addition, the mutant was obtained from the Chlamy collection long ago and we kept it at -155 deg. C all the time.

Major remarks:
1- The method section is rather limited in explanations and description of the experimental procedure. The manuscript would gain a lot in clarity of the experimental procedures by elaborating more on what has been done, especially for quantification of proteins and quantifying the PSII absorbance cross section.
Answer: Thank you for the suggestion we have now updated M&M and especially the PSII rate quantification ("PSII antenna size measurements") is now described in more detail.

2- A lot of emphasis is put on protein quantification using western blot. However, such method has to be analysed with a lot of cautious and require many controls that are not shown in the present manuscript. For example, normalizing to an internal loading control (like actin or tubulin) should have been done; absence of saturation of the signal should have been ensured... Clarification of the western blot-based quantification would help in this manner. Please provide raw data with molecular weight ladder if possible to improve reproducibility of the results.
Answer:In all immunoblots reported we have loaded the same amount of protein and we have now added the ponceau staining picture of all blots.

3- The title is a bit misleading here since it gives the impression that the authors have revealed the mechanistic role of LHCBM1. While they only propose a mechanism for LHCBM1 role, they have shown that lack of NPQ in npq5 is not due to low LHCSR3 amount but rather to its capacity to respond to pH. Thus a title like "LHCBM1 tunes the proton response of LHCSR3-mediated NPQ" would be more appropriate.
Answer: We have modified the title into "LHCBM1 tunes the response of LHCSR3-mediated non-photochemical quenching in Chlamydomonas"

Minor remarks:
The authors show that the level of LHCSR1 is strongly impacted in npq5 mutant, it would be nice to have the authors' view on this effect in the discussion. Could the lack of LHCBM1 completely destabilize LHCSR1 protein and lead to its degradation? Would LHCBM1 also be the main docking site of LHCSR1?
There is very little LHCSR1 in these cells and we do not think we can reach any solid conclusions about the effect of LHCBM1 on it and we prefer not to speculate.

Please add lines number to facilitate remarks.
Done

"A quickly activated photoprotection mechanism": NPQ is not necessarily quickly activated. Please consider revising

We have changed the sentence as suggested

" expressed upon exposure of the cells to high light": LHCSR3 has recently been shown to be also importantly controlled by CO2 levels, which presumably would be the main factor (https://doi.org/10.1101/2021.10.23.465040) , please also consider citing more recent analysis like (https://doi.org/10.1101/2021.10.23.463292).

We have changed the sentence and added the two references

At some places, the authors are using "*" to separate between units, like "photons*m-2 *s-1"; please consider changing the "*" by a space, like in Fig. 2C. Please check that the formatting used in consistent throughout the manuscript.

Done

When showing histograms or bar plots, please show explicitly each biological replicate, since for small data sets ($n<10$), it is easier to visualize for the reader and more transparent than +-SEM or +-SD. Please also provide statistical analysis on the data whenever possible

We have added the statistical analysis as suggested

"lower PSI/PSII ratio than the control strain.": Please provide a reference to a figure here

Done

"The cells respond to the reduced PSII functionality by lowering the amount of PSI to restore a balance between the two photosystems.": Please provide a reference to a figure here

We have eliminated this sentence as suggested by reviewer 1

28-Apr-2023

Prof. Dr. Roberta Croce
Vrije Universiteit Amsterdam
Amsterdam, Netherlands 1081 HV
Netherlands

RE: LHCBM1 tunes the response of LHCSR3-dependent non-photochemical quenching in Chlamydomonas

Dear Dr. Roberta Croce:

We are pleased to accept your manuscript for publication in the Plant Physiology. This acceptance is contingent on revision based on the comments of our reviewers. In particular, please consider the following:

The issue raised by reviewer 1 regarding the use of Fv/Fm as a measure of the QE of PSII, the rationale and caveats could be included directly in the MS to highlight these concerns and arguments. The second reviewer has raised a number of minor points including the inclusion of raw data pictures for western blots with molecular ladders if possible. There are also some aspects of the Fig 2 that need to be amended.

Please highlight all changes and include a detailed annotation to changes to the text, with line numbers, and noting your responses to the comments.

To submit your revised manuscript, click:

Link Not Available

If you cannot return the revised manuscript within 8 weeks of receipt, please let us know. Otherwise, we will assume that you have elected not to revise the manuscript and are withdrawing it.

Thank you for allowing us to review your work. We look forward to hearing from you soon.

Sincerely,

Plant Physiology Board of Editors

-- Reviewer comments:
Reviewer #1 (Comments for the Author):

The authors satisfactorily amended their manuscript, responding to my critical comment - except one point.
Namely, Fv/Fm cannot and should not be equated with the maximum quantum effiiciency of PSII. They argue that but "if a saturating pulse and not a single-turnover flash is used for the experiments, it is a close proxy of the QE". This argument overlooks the fact that - as shown by Sipka et al. 2019 Physiol Plant, 2021 Plant Cell - the consecutive flashes after the first STSF, which lead to the F1 (Of course, qualitatively Fv/Fm can still be used to characterize the photochemical activity (and structural dynamics of PSII), and thus - I think - it won't concern the main conclusions of the authors. Nonetheless, the authors should avoid its erroneous assignment.

Reviewer #2 (Comments for the Author):

The authors have adequately answered most of my concerns.
I still have some minor comments to the manuscript:
1: Since the authors could not get an independent lhcbm1 mutant or a complementing strain, it would be important to check that the presence of Arginine in the growth media of the WT control is not responsible for some of the differences observed. Although this is unlikely to change the main conclusions of the manuscript related to NPQ, arginine affects the physiology of the algae and hence the electron transport which could change turnover rates of photosystems and affect substantially the conclusions lines 183-205.
2: The authors claim in their response to have added a ponceau picture of all blots while only one ponceau appears in the manuscript in Fig. 1 A. I would also like to reiterate the demand to have all raw data pictures for western blots with molecular

ladders if possible, to improve reproducibility of the results by others.
3: Fig. 2 A: Single data points and average are inconsistent for the WT at 24h. There are either data points missing, or the average should be different. Please correct. Many figures are heavily pixelated.
A side note on Fig. 2: While bars are vectorial, single data point and letters are rasterized images (and very pixelated). Please homogenize.

Response to the reviewers' comments

Reviewer #1 (Comments for the Author):
The authors satisfactorily amended their manuscript, responding to my critical comment - except one point. Namely, Fv/Fm cannot and should not be equated with the maximum quantum effiiciency of PSII. They argue that but "if a saturating pulse and not a single-turnover flash is used for the experiments, it is a close proxy of the QE". This argument overlooks the fact that - as shown by Sipka et al. 2019 Physiol Plant, 2021 Plant Cell - the consecutive flashes after the first STSF, which lead to the F1 (<Fm) to Fm increment (whichh can be quite substantial), induce no additional stable charge separations, and thus cannot be related to the QE of PSII.
Of course, qualitatively Fv/Fm can still be used to characterize the photochemical activity (and structural dynamics of PSII), and thus - I think - it won't concern the main conclusions of the authors. Nonetheless, the authors should avoid its erroneous assignment.

Response: Following the suggestion of the reviewer, we have modified the definition of Fv/Fm. We no longer mention that it represents the maximum PSII quantum efficiency; instead, we now describe it as "a proxy for the status of PSII".

Reviewer #2 (Comments for the Author):

The authors have adequately answered most of my concerns.
I still have some minor comments to the manuscript:
1: Since the authors could not get an independent lhcbm1 mutant or a complementing strain, it would be important to check that the presence of Arginine in the growth media of the WT control is not responsible for some of the differences observed. Although this is unlikely to change the main conclusions of the manuscript related to NPQ, arginine affects the physiology of the algae and hence the electron transport which could change turnover rates of photosystems and affect substantially the conclusions lines 183-205.

We apologize, the description in the Materials & Methods section was unclear. Arginine was used exclusively with TAP medium and not with HRM. This means that the NPQ experiments were conducted without it. We have clarified this in the M&M section.

2: The authors claim in their response to have added a ponceau picture of all blots while only one ponceau appears in the manuscript in Fig. 1 A. I would also like to reiterate the demand to have all raw data pictures for western blots with molecular ladders if possible, to improve reproducibility of the results by others.

We have now added all the Ponceau images to Figure S2 for all biological replicates. Unfortunately, the quality of the Ponceau images is not optimal, but the loading is discernible. The thylakoid proteins serve as our "ladders" because their mobility on these types of gels is well-known. They represent a more suitable ladder than the commercial ones, given that the mobility of membrane proteins in SDS-PAGE differs substantially from that of soluble proteins.

3: Fig. 2 A: Single data points and average are inconsistent for the WT at 24h. There are either data points missing, or the average should be different. Please correct. Many figures are heavily pixelated. A side note on Fig. 2: While bars are vectorial, single data point and letters are rasterized images (and very pixelated). Please homogenize.

Thank you for noticing this. We have corrected the figure.

23-Sep-2023
Prof. Dr. Roberta Croce
Vrije Universiteit Amsterdam
Amsterdam, Netherlands 1081 HV
Netherlands

MSID: PP2023-RA-00234DR1
MS TITLE: LHCBM1 tunes the response of LHCSR3-dependent non-photochemical quenching in Chlamydomonas

Dear Dr. Roberta Croce:

I am pleased to inform you that your revised manuscript "LHCBM1 tunes the response of LHCSR3-dependent non-photochemical quenching in Chlamydomonas" has been accepted for publication in Plant Physiology. Your manuscript will be checked for consistency with journal formatting and image standards by one of our Science Editors, who will contact you shortly. They may have a number of queries and suggestions for you. Please keep in mind that they work to increase the online visibility of your article and its attraction for readers from the widest of backgrounds. You will have the opportunity to review any revisions, and I hope you will work constructively with them to get the most out of your research. You will also have the opportunity to review all changes when you receive the author proofs for your article.

Once validated for publication, your article will be sent to a Science Editor for a final review of your manuscript and source files. After your final files are uploaded, your article will appear in the next available issue and the online Preview version of your article will be posted after your signed Copyright and Checklist forms and final source files are received.

Please be sure to include the following in your manuscript file:
Harvard style citations
List of all author contributions and funding information at the beginning of manuscript after the titles and author list.
List all supplemental materials associated with the article, with titles, at the end of the ms, before the references and acknowledgments section.
Complete Figure Legends for all figures appearing in the article listed before the Literature Cited.

Thank you and congratulations on your Plant Physiology paper!

Sincerely,
Plant Physiology Board of Editors

=====
FIRST AUTHOR PROFILES
=====

We recognize the hard work that goes into being First Author of a Plant Physiology paper by publishing a "First Author Feature" for each First Author alongside your paper.

If the first author(s) would like to submit information for a feature on Facebook and Twitter, fill out the form by clicking on the link below:

Plant Physiology First Author Feature Form: https://forms.office.com/r/ZBCGZfhm8F
If you cannot access the form through the link above, please contact Rachel at rbelsky@aspb.org.

To upload an image of yourself to accompany your profile, please put your photo in the correct journal folder at the link below. You must label your image: Author First Name Last Name Article Title.

First Author Profile Image Dropbox: https://www.dropbox.com/scl/fo/xz029p7qjr2jml2rp2kmq/h?dl=0&rlkey=6fkxcaqmxx35ap152b2osy1fz

=====
IMPORTANT REMINDER: PEER REVIEW REPORTS
=====

If you opted to publish a peer review report along with your article during the original submission process, it will be prepared by the editorial staff and publicly posted with your manuscript, inside the zip file that contains any other supplemental material. As a reminder, the peer review report is a public record of all comments from editors and reviewers, as well as your prior responses, as you received them in the decision letters for each draft of your manuscript. If you agreed to publish this report and have changed your mind, or are not sure if you selected this option, please contact the editorial office as soon as possible before signing the license agreement from our publisher.

-- Reviewer comments:
